# Levofloxacin-Induced Acute Hyperpigmentation Changes in a Chronic Kidney Disease Patient

**DOI:** 10.1155/2020/6186471

**Published:** 2020-11-07

**Authors:** Shakuntala S. Patil, Sachin M. Patil, Ryan Campbell, Manisha Singh, Matthew Plotkin

**Affiliations:** University of Missouri Hospital and Clinic, 1 Hospital Drive, Columbia, MO 65212, USA

## Abstract

Medication-induced cutaneous hyperpigmentation has variable clinical presentations and is dependent on the specific drug involved. Most commonly, an attentive patient observes such changes early in the course; when missed by the patient, such changes are usually noted by an observant clinician. Clinical diagnosis can be challenging if the patient is on multiple medications because other causes must be excluded. This condition occurs via multiple mechanisms. Frequently, the pigmentary change is reversible with discontinuation of the drug. Causative medications include nonsteroidal; anti-inflammatory agents, antimalarials, antibiotics, psychotropics, amiodarone, and chemotherapeutic agents. The; antimicrobials responsible for hyperpigmentation are antimalarials, tetracyclines, tigecycline, dapsone, rifampicin, and antiretrovirals such as zidovudine. Sunlight exposure can worsen the pigmentation seen with some of the above antimicrobials (e.g., dapsone). Here, we describe an older adult white woman presenting with acute cutaneous; hyperpigmentation of the bilateral lower extremities while on levofloxacin therapy. Hyperpigmentation resolved after cessation of the agent. Our case highlights this unique acute presentation after only a few days of oral levofloxacin.

## 1. Introduction

Fluoroquinolones are a class of antibiotics that belong to the quinolone family of antimicrobial agents. Fluoroquinolones have good oral bioavailability, excellent tolerability, and a broad spectrum of activity. Some fluoroquinolones are not in current clinical use due to their uncommon toxicities. The ones commonly used in clinical practice are ciprofloxacin, levofloxacin, and moxifloxacin. They stop bacterial deoxyribonucleic acid synthesis via the inhibition of deoxyribonucleic acid gyrase and topoisomerase IV. Ciprofloxacin and levofloxacin are indicated in the treatment of urinary tract infections. Accordingly, they constitute some of the more commonly prescribed oral antibiotics [1]. The most common adverse effects are reported in the gastrointestinal tract (nausea, vomiting, and diarrhea), nervous system (headache, insomnia, mood alterations, hallucinations, and delirium), and connective tissue (tendinitis, retinal detachment, and aortic dissection). Although uncommon, skin reactions typically include rashes [1]. Upon review of the medical literature in PubMed, only four cases of levofloxacin-induced cutaneous hyperpigmentation have been reported [2–5]. In most of these cases, the duration of levofloxacin was more than one week, and typically on the order of months. We report a case of bilateral lower extremity hyperpigmentation that occurred in an older white woman with Stage 5 chronic kidney disease (CKD) after she completed a five-day course of oral levofloxacin for cystitis.

## 2. Case Report

**Figure 1:**
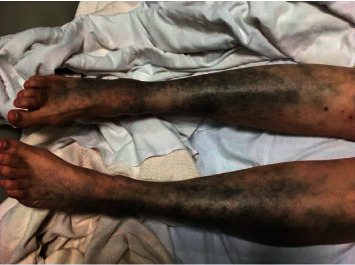
Bilateral legs with pigmentary changes.

**Figure 2:**
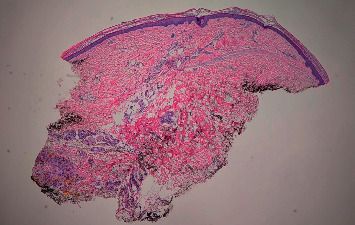
Low-power magnification shows widely dispersed dermal pigmentation and an epidermis with no significant pathologic changes (20x; hematoxylin and eosin staining).

**Figure 3:**
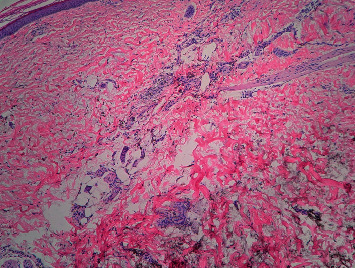
Intermediate magnification shows coarse cytoplasmic granular brown pigmentation within aggregates of macrophages surrounding eccrine structures in the dermis (100x; hematoxylin and eosin staining).

**Figure 4:**
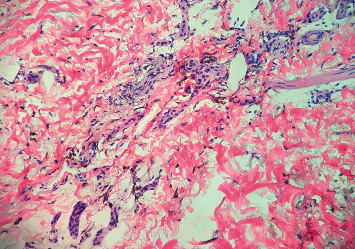
Intermediate magnification shows coarse cytoplasmic granular brown pigmentation within aggregates of macrophages surrounding eccrine structures in the dermis (200x; hematoxylin and eosin staining).

**Figure 5:**
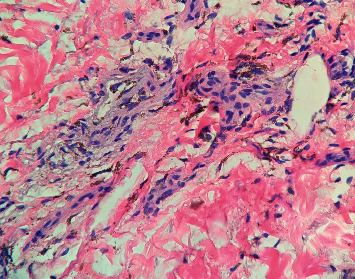
High-power magnification displays coarse brown pigmented cytoplasmic granules within the dermal macrophages (400x; hematoxylin and eosin staining).

A 58-year-old white woman presented to the clinic with a hyperpigmented bluish-grey rash over the bilateral lower extremities persistent for two days (Figure 1). Past medical history was significant for autosomal dominant polycystic kidney disease with Stage 5 CKD and hypertension. She was not on any anticoagulation. A few days before this clinic visit, she was treated with oral levofloxacin 750 mg daily for five days (total of 3750 mg) for a urinary tract infection. She denied any recent sick contacts or fever. On further questioning, she admitted to noticing this rash on day three of treatment. She denied any significant sun exposure to her lower extremities. Physical examination revealed a nonblanching bluish-grey rash located on the bilateral lower extremities, especially over the anterolateral aspect involving bilateral feet. Distal pulses were regular. No other significant clinical findings were observed on examination. Her blood work revealed a healthy complete blood cell count with differential. The basic metabolic panel revealed a stable baseline creatinine. She was referred to the dermatology clinic where she underwent skin biopsy of the lesions on her lower extremities. Skin biopsy revealed scattered deposits of brown-black pigment in dermal spindle cells and minimal inflammation, indicating pigmentary rash (Figure 2). High-power magnification displayed coarse brown pigmented cytoplasmic granules within the dermal macrophages (Figures 3–5). The patients' medication history revealed no new medication other than the levofloxacin over the last four weeks. The patient had completed antibiotic therapy when she presented to the clinic with hyperpigmentation changes. On follow-up at the clinic three weeks later, the rash seen earlier over the bilateral lower extremities had entirely resolved with no residual changes.

## 3. Discussion

Levofloxacin is a well-tolerated medication with good oral bioavailability, making it an optimal choice as an oral antibiotic. Levofloxacin is associated with dermatologic adverse effects in less than 3% of instances [1]. The most common presentation is an unspecified rash. With a treatment duration of five days or fewer, such a rash is infrequent [1]. Uncommonly, it can cause a drug reaction with eosinophilia and systemic symptoms syndrome, phototoxicity, Stevens—Johnson syndrome, fixed drug eruption, and leukocytoclastic vasculitis [5]. It can cause hyperpigmentation, albeit rarely. A small but significant fraction (10% to 20%) of acquired pigmentation cases are drug-induced [6]. Minocycline is the most common antibiotic responsible for drug-induced hyperpigmentation [6, 7]. In a few isolated cases, polymyxin and tigecycline have caused this condition [7]. Among the quinolones, few cases have been reported, and all were due to pefloxacin and levofloxacin. The levofloxacin tablet coating contains small quantities of iron oxide and hydroxide [3]. The mechanisms responsible for this hyperpigmentation include drug metabolite deposits, precipitation of intracellular iron chelate complexes within macrophages, and increased intracellular melanin production [4, 6, 8]. A review of the medical literature via PubMed revealed only four reported cases of levofloxacin-induced cutaneous hyperpigmentation—all confirmed by biopsy [2–5]. On review of these cases, three patients were on oral levofloxacin therapy for more than four weeks [2, 3, 5]. One case report mentioned the use of levofloxacin for only eleven days. This patient had significant exposure to sunlight with phototoxicity [4]. None of the four case reports mentioned any significant CKD. In our patient, the pigmentary changes on the lower extremities were seen on the third day of the five day therapeutic regimen. She received a higher dose of levofloxacin (a total of 3750 mg) based on her renal function. Levofloxacin and its metabolites are renally excreted. A higher dose in a patient with unhealthy renal function results in the accumulation of the drug and its metabolites. Her prior Stage 5 CKD predisposed her to a higher risk of developing adverse effects. The standard levofloxacin dose in Stage 5 CKD is a 500 mg bolus dose followed by 250 mg every 48 hours over five days (for a total of 1000 mg). Based on the renal calculation of the dose, our patient received a total of 31 days' worth of levofloxacin over five days. Thus, our Stage 5 CKD patient received a significantly higher dose of levofloxacin (3750 mg instead of 1000 mg for five days), resulting in the accumulation of the drug and its metabolites. The rash resolved completely in three weeks after discontinuation of the drug. Discontinuation of the offending medication is currently the only therapeutic modality for levofloxacin-induced hyperpigmentation. Pigmentary changes in all the case reports resolved following the discontinuation of levofloxacin [2–5].

## 4. Conclusion

We report an unusual adverse effect of levofloxacin in a CKD patient. This case report stresses the importance of dose adjustments of renally excreted medications in the CKD population. Also, CKD patients may present with pigmentary changes earlier than a typical patient due to supratherapeutic drug levels. While such changes are reversible by discontinuation of the drug, in such instances, a new regimen based on the antibiogram must then be considered.

## Data Availability

No data were used to support this study.
